# MD-Former: Multiscale Dual Branch Transformer for Multivariate Time Series Classification

**DOI:** 10.3390/s25051487

**Published:** 2025-02-28

**Authors:** Yanling Du, Shuhao Chu, Jintao Wang, Manli Shi, Dongmei Huang, Wei Song

**Affiliations:** 1 College of Information Technology, Shanghai Ocean University, Shanghai 201306, China; yldu@shou.edu.cn (Y.D.); m220901568@st.shou.edu.cn (S.C.); 2052307@st.shou.edu.cn (M.S.); 2 College of Marine Living Resource Sciences and Management, Shanghai Ocean University, Shanghai 201306, China; jtwang@shou.edu.cn; 3 College of Electronics and Information Engineering, Shanghai University of Electric Power, Shanghai 201306, China; dmhuang@shou.edu.cn

**Keywords:** multivariate time series classification, transformer, multiscale architecture, UEA datasets

## Abstract

Multivariate Time Series Classification (MTSC) is a challenging task in real-world applications. Current approaches emphasize modeling multiscale relationships over time. However, the Multivariate Time Series (MTS) also exhibits multiscale cross-channel relationships. Furthermore, the long-term temporal relationships in time series are difficult to capture. In this paper, we introduce MD-Former, a Multiscale Dual-Branch Attention network leveraging the Transformer architecture to capture multiscale relationships across time and channels for MTSC. In MD-Former, MTS is embedded into 2D vectors using Channel-Patching (CP) to retain channel information. Following this, we develop two branches: the Interlaced Attention Branch (IAB) and the Channel-Independent Attention Branch (CIAB). The IAB facilitates the fusion of information across channels and time, while the CIAB prevents the loss of information resulting from excessive fusion. Both the IAB and CIAB consist of multiple layers, each representing a distinct time scale. Finally, we utilize features from each layer of both IAB and CIAB as inputs to the Multiscale Classification Head (MCH) for feature fusion and classification. Experimental results show that MD-Former achieves performance levels that are comparable to SOTA methods in MTSC.

## 1. Introduction

Time series data, characterized by sequential recording and high temporal correlations, are categorized into univariate time series (UTS) and multivariate time series (MTS). MTS are particularly well suited for modeling complex real-world phenomena involving multiple variables, such as ship trajectories, represented in speed, direction, longitude, and latitude. Multivariate time series classification (MTSC) aims to identify patterns in multivariate time series data. It is of great importance in various real-world applications, including structural health monitoring [1], medication tampering monitoring [2], disease diagnosis [3], fault location in power systems [4], and abnormal event detection in industrial devices [5].

In the early days, models based on linear assumptions [6] and Markov processes [7] were widely applied to MTSC problems. However, as the complexity of time series data increased, their effectiveness became limited. Meanwhile, distance-based similarity methods have also been used for MTSC tasks, but their transferability performance is limited. Advances in deep learning have made deep learning-based MTSC methods a focal point for researchers. In contrast to conventional approaches, deep learning-based methods demonstrate superior performance in handling complex data, which can directly map raw data to high-dimensional features without requiring extensive domain knowledge. Previous studies have demonstrated the significant success of Convolutional Neural Networks (CNNs) in Multivariate Time Series Classification (MTSC) tasks [8,9,10]. However, CNNs face challenges in modeling long-term temporal dependencies, as they are more adept at capturing local relationships while struggling to effectively model global dependencies. On the other hand, global dependencies over long sequences are essential for uncovering the underlying patterns in the data. Long Short-Term Memory (LSTM) networks are often considered more suitable for handling time series data, which has led many methods to utilize LSTM [11]. However, LSTM also has limitations, especially when dealing with long sequences, due to issues like vanishing and exploding gradients. These challenges can result in Recurrent Neural Networks (RNNs), including LSTMs, performing worse than CNNs in some cases. Conversely, Transformer models have demonstrated tremendous potential in time series analysis, largely due to their powerful global modeling capabilities [12,13].

Although these deep learning-based methods have achieved promising results, they often focus solely on capturing temporal dependency while overlooking cross-channel dependency. SVP-T [14] attempts to model both time and channel relationship simultaneously, yet falls short in extracting multiscale features. In the context of time series (TS) data, multiscale features are essential for identifying periodic patterns at various time spans (e.g., seconds, minutes, and hours), thereby enhancing the analysis of temporal patterns in the data. Moreover, the data embedding approaches in current MTSC methods mostly involve directly inputting into linear layers. However, in the field of MTS forecasting, many studies suggest that the embedding approach notably influences the model’s representational capacity [15,16]. Additionally, while certain techniques strive to capture cross-channel dependencies, an excessive fusion of channels may result in information degradation, underscoring the importance of preserving the channel-independent branch.

Recent advances in Transformer-based models highlight their tremendous potential in global time modeling. However, in the MTSC domain, advanced models still have three main limitations: (1) insufficient joint modeling of cross-channel and cross-time dependencies; (2) neglect of multiscale periodic structures; (3) suboptimal embedding strategies—either excessively mixing channel information or neglecting subsequent channel-independent designs. These limitations have driven us to develop a unified framework to address these challenges.

To effectively leverage time, channel dependencies, and multiscale information, we propose MD-Former, a novel multiscale dual-branch MTSC method based on Transformer. Specifically, our approach involves the utilization of a channel-independent embedding, referred to as Channel-Patching (CP), to ensure the preservation of information independence across channels. Then, we introduce Multiscale Dual-Branch Attention (MDBA) to capture multiscale information, encompassing the Interleaved Attention Branch (IAB) and the Channel-Independent Attention Branch (CIAB). Each layer of MDBA corresponds to periodic patterns at varying time spans. Among them, the IAB is employed to capture cross-channel and cross-time dependencies, while the CIAB ensures information isolation to prevent information loss resulting from excessive channel fusion. Finally, a Multiscale Classification Head (MCH) is designed to aggregate features from multiple layers and produce the ultimate classification outcomes. The main contributions of this paper are as follows:

- We propose the MD-Former, a Transformer model designed to incorporate cross-channel, cross-temporal dependencies and multiscale information for MTSC. This model presents a unique strategy for tackling MTSC challenges.
- We introduce a novel mechanism called MDBA, in which IAB alternates between time and channel attention to integrate channel and temporal information. The CIAB is specifically dedicated to modeling temporal relationships while maintaining channel independence to prevent information loss from excessive fusion. The multiscale architecture effectively leverages the inherent periodic relationships of time series data.
- Experimental results show that MD-Former exhibits significant classification performance on the popular public dataset UEA [17]. Among 12 comparison methods, the MD-Former wins on nine datasets and achieves first place in the metrics of AVG acc, AVG rank, and MPCE.

## 2. Related Work

### 2.1. Multivariate Time Series Classification

Time series data mining is recognized as one of the top ten most difficult challenges within the realm of data mining [18] where MTSC plays a pivotal role. Traditional time series analysis has predominantly relied on well-established statistical methods such as the ARIMA model [6] and spectral density estimation [19] to capture temporal dependencies. These approaches offer a robust theoretical foundation for analysts, enabling the modeling of linear relationships and periodic behaviors under the assumption of stationarity. However, they exhibit clear limitations when applied to multivariate contexts. ARIMA and similar classical models are primarily designed for univariate forecasting. Though extensions like the VARMA model [20] can capture some interdependencies in multivariate data, they struggle with high-dimensional, nonlinear relationships. Furthermore, while spectral methods can offer insights into periodic components, they assume stationarity and linearity, which do not align with many real-world datasets. As such, these methods face difficulties in detecting complex, long-term dependencies across multiple channels. In light of these challenges, researchers have turned to alternative approaches.

MTSC methods can now be broadly categorized into traditional model-based methods, instance-based methods, and feature-based methods [21]. Traditional model-based methods primarily rely on the Markov model and its variants [7,22,23]. However, the assumption of the Hidden Markov Model is overly simplistic and cannot effectively handle complex data, particularly the long-term dependency relationships in time series. Instance-based methods primarily aim to find a suitable way to compare MTS data, usually employing distance metrics for similarity measurement [24,25,26]. However, similarity-based methods often suffer from poor transferability across datasets due to their failure to capture the feature dependencies between data points. Feature-based methods, on the other hand, excel in handling high-dimensional complex data and demonstrate superior transferability performance. Among them, deep learning has gradually become the mainstream approach for MTSC.

Deep learning methods based on feature extraction and task-oriented approaches have achieved significant success. The CNN, benefiting from its powerful capability to extract local features, has shown promising results. For example, MVCNN [8] introduces an innovative CNN structure tailored for the analysis of multivariate time series data, emphasizing the interrelationships and co-movements among a set of temporal variables. InceptionTime [9] endeavors to incorporate the Inception architecture into time series data to capture multiscale information. ShapeNet [10] embeds shapelet candidates of varying lengths into a unified space for shapelet selection, yet it neglects positional details within the shapelet. Although CNN methods have demonstrated effectiveness in MTSC, they frequently encounter challenges in capturing long-term dependency relationships that are essential for analyzing time series data. In response to this limitation, researchers have shifted their attention towards models better suited for sequential data, such as LSTM. The multivariate LSTM-FCN [11] model aims to adapt successful univariate time series classification models, including LSTM-FCN and ALSTM-FCN, to MTSC tasks, enhancing accuracy through the incorporation of SE Blocks.

### 2.2. Transformer-Based Methods

The Transformer addresses the limitations of sequence models such as RNN/LSTM, such as the vanishing gradient problem on long sequences. It demonstrates significant potential on long sequences and has achieved great success in various fields, such as natural language processing [27,28] and vision [29,30,31,32].

In recent years, researchers have begun to focus on the application of the Transformer in MTSC, yielding encouraging outcomes. TapNet [33] introduces attention prototype networks into MTSC and extends them to semi-supervised learning by leveraging unlabeled data. ConvTran [34] endeavors to investigate the efficacy of positional encoding in time series and introduces a unique absolute positional encoding technique tailored for time series data, named tAPE, in addition to an efficient implementation of relative positional encoding (eRPE) computation. Then, ConvTran proposes a combination of tAPE, eRPE, and convolution-based input encoding to enhance the capacity for capturing positional information and enhancing data embedding. TST [35] uses an unsupervised pretraining task specifically designed for time series data to enhance the model’s representation capability. TARNet [36] further introduces a data-driven masking strategy, where crucial data for downstream tasks are masked and reconstructed, thereby improving the model’s representation capability on the downstream tasks. Research on the Transformer has shown that it lacks sufficient local perception. Therefore, FMLA [13] enhances locality awareness through layer-wise interactions with deformable convolutional blocks and online knowledge distillation. DA-Net [12] introduces a novel network based on dual attention for exploring both local and global features in multivariate time series classification.

Recent research findings suggest that embedding methods and modeling cross-channel dependencies are important issues for multivariate time series data. PatchTST [15] introduces the concept of patching into MTS for the first time and adopts a channel-independent design, achieving significant results. SVP-T [14] also divides multivariate time series data into subsequences and models the dependencies in time and channel using a VP layer. Most similar to our work is Crossformer [16], which attempts to segment multivariate time series data, model the relationships in time and channel through a two-stage attention mechanism, and design a multilayer Encoder–Decoder to complete the MTSF task. We also adopt a two-stage attention mechanism, but we introduce channel-independent branches and redesign data embedding, multiscale attention, and classification heads to better adapt to the MTSC problem.

Although existing MTSC methods perform well, there are still shortcomings in capturing dual dependencies across time and channels and exploring multiscale relationships. To address this issue, our proposed method focuses on how to capture dependencies across channels and time, as well as modeling multiscale relationships that demonstrate different periodicities in multivariate time series data.

## 3. Methodology

**Figure 1:**
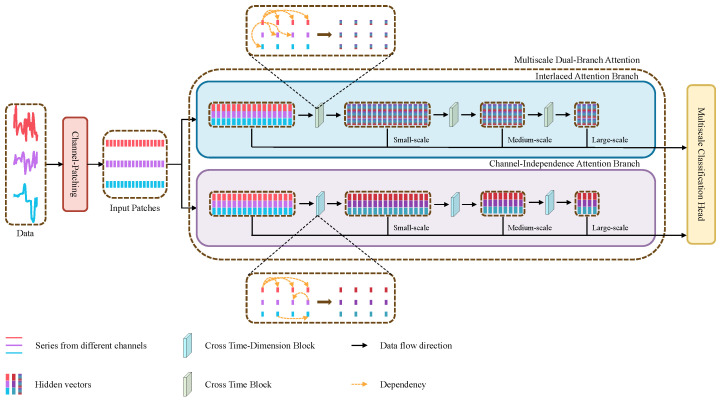
The overall framework of MD-Former.

To comprehensively explore the interrelated dependencies at various scales over time and channels within time series data, we present the MD-Former, thee overall structure of which is illustrated in Figure 1. In this section, we first define the MTSC problem and introduce an overview of the MD-Former network followed by details of the key components.

### 3.1. Problem Definition and Model Structure

We consider the following problem: we are given the multivariate time series data

$$X=\left\{X_{1},X_{2},X_{3},\ldots ,X_{L}\right\}\in R^{L\times C}$$

where each $X_{i}$ consists of *C* channels (for multivariate time series, C>1), and *L* represents the length of *X*. Each training sample comes with a unique label *y*, which represents an integer class ID for the classification task. Utilizing these training samples, a model is constructed to forecast the label $\hat{y}$ of unseen data $\hat{X}$.

The overall framework of MD-Former is depicted in Figure 1, showing the model’s three primary components: Channel-Patching (CP), Multiscale Dual-Branch Attention (MDBA), and Multiscale Classification Head (MCH). CP preprocesses the data by segmenting the multivariate time series into patches to align with the input requirements of MDBA. MDBA is composed of two branches, the Interlaced Attention Branch (IAB) and the Channel-Independent Attention Branch (CIAB), each comprising multiple layers. The IAB is responsible for extracting and integrating features across channels and time. The CIAB aims to maintain channel independence to capture pure temporal dependency. Finally, the MCH aggregates the multiscale features from MDBA to generate the final classification results.

### 3.2. Channel-Patching

Recent research has highlighted the effectiveness of patching in handling time series data and the importance of channel independence for feature extraction, even though it may not seem immediately intuitive [15]. To enable the feature extraction network to obtain channel-independent data embeddings for extracting pure temporal relationships while maintaining a channel-independent branch, we propose Channel-Patching, a channel-independent data embedding method.

**Figure 2:**
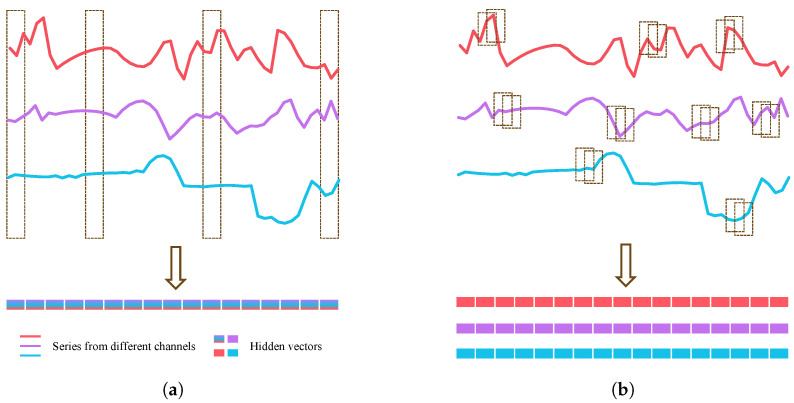
Illustration for Channel-Patching: (**a**) Embedding method of previous Transformer-based models: all channels at the same time step are embedded into a vector. (**b**) Channel-Patching: in different channels, neighboring time steps are embedded into a vector, either overlapping or non overlapping.

As shown in Figure 2b, we propose a channel-independent embedding method to ensure the channel independence during the data embedding phase. Previous methods typically combine all channels at the same time step into a single vector, thereby forming a one-dimensional sequence (as shown in Figure 2a). Channel-Patching (CP) processes each channel separately, embedding overlapping or non overlapping adjacent time steps into a vector, thus generating a two-dimensional matrix that preserves the cross-channel relationships between different time steps. It is important to note that CP does not allow the independent selection of overlapping or non overlapping embedding strategies for specific channels; the same strategy is applied to all channels.

**Figure 3:**
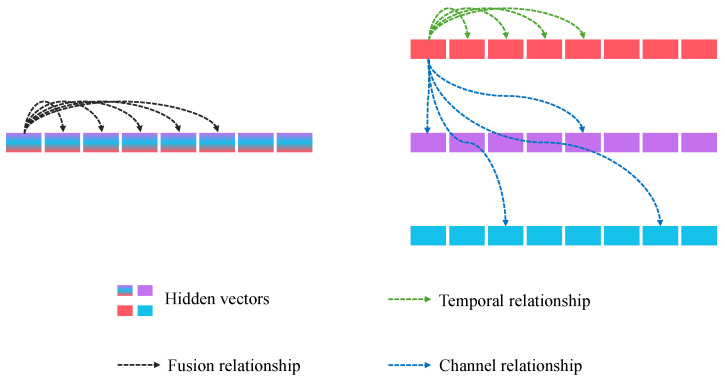
Relationship modeling of different data embedding methods.

As shown in Figure 3, the traditional patching data embedding method generates a one-dimensional vector, which has already integrated the channel relationships. In subsequent modeling, only the fused relationships across different time steps can be modeled. However, the premature integration of channel relationships has some impact on the modeling of temporal relationships. Channel-Patching generates a two-dimensional matrix during the data embedding phase, allowing the modeling of cross-channel relationships between different time steps in subsequent feature modeling. Additionally, a dual-stage strategy can be employed to freely choose whether to model temporal or channel relationships first. In MD-Former, we choose to model temporal relationships first, as they are the most crucial attribute of time series data. Integrating channels first may potentially affect the model’s ability to extract pure temporal relationships.

Specifically, for a multivariate time series $X\in R^{L\times C}$, we first ensure channel independence by decomposing it into univariate time series $X=\{X^{1},X^{2},X^{3},\ldots ,X^{C}\}$ and then partitioning $X^{i}\in R^{L\times 1}$ into overlapping or non overlapping patches. The length of a patch is denoted by *K* and the stride by *S*. Thus, a sequence of length *L*, $X^{i}$, can be divided into *P* patches, where $P=\left\lfloor \frac{L-K}{S}\right\rfloor +1$, and $X^{i}\in R^{P\times 1}$, $X\in R^{P\times C}$. After the patching process, the model input is reduced from *L* to approximately $\frac{L}{S}$. This reduction results in a quadratic decrease in memory usage and computational complexity with respect to *S*. Therefore, Channel-Patching allows the model to observe longer time series within the constraints of training time and GPU memory, which can potentially improve model performance.

CP ensures the independence of data across channels, avoiding channel mixing during the data embedding stage. Premature channel fusion during temporal relationship modeling may compromise the integrity of temporal information. Additionally, CP serves as a prerequisite for implementing the dual-branch structure.

### 3.3. Multiscale Dual-Branch Attention

Multiscale Dual-Branch Attention (MDBA) is the core mechanism of MD-Former, consisting of two branches: the Interlaced Attention Branch (IAB) and the Channel-Independent Attention Branch (CIAB).

#### 3.3.1. Interlaced Attention Branch

**Figure 4:**
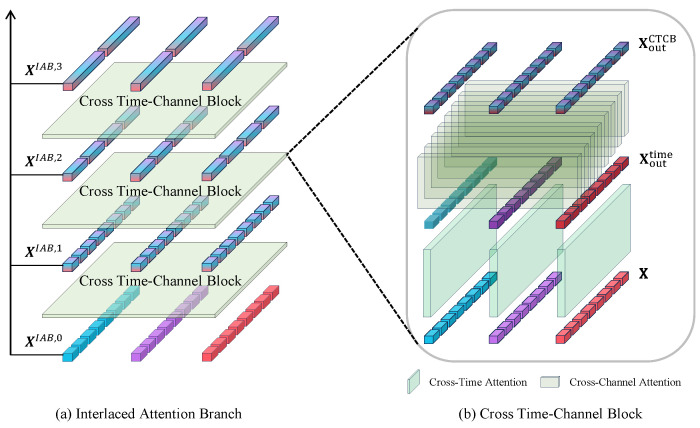
Interlaced Attention Branch.

As shown in Figure 4, the IAB comprises multiple Cross Time–Channel Blocks (CTCBs) arranged in a stacked configuration. Each CTCB consists of a fusion of time and channel attention layers.

When considering an input $X\in R^{L\times C}$, it can be directly unfolded into a 1D sequence to adapt to the input of the self-attention mechanism, similar to the approach used by ViT [29] for image processing and PatchTST [15] for time series. However, this operation leads to a significant increase in computational and parameter complexity, especially for processing long-sequence data. Therefore, we adopt a dual-stage strategy, first modeling the primary attribute (temporal relationships) and then modeling channel relationships. This is achieved through stacked dual attention layers to capture dependencies across time and channels.

In a CTCB, two layers of Multihead Self-Attention (MSA) are utilized to capture dependencies across time and channels. In a 2D input $X_{t,c}\in R^{P\times C}$, where *t* denotes time, *c* denotes channel, and *P* is the number of patches following the previous layer, $X_{:,c}$ represents vectors across all time steps for channel *c*, and $X_{t,:}$ represents vectors across all channels for time step *t*. A CTCB can be conceptualized in two distinct stages:

(1) $$\left\{\begin{array}{c} X_{MSA}^{time}=LN_{1}\left(X_{:,c}+Dropout\left(MSA^{time}\left(X_{:,c},X_{:,c},X_{:,c}\right)\right)\right)\\ X_{out}^{time}=LN_{2}\left(X_{MSA}^{time}+Dropout\left(MLP^{time}\left(X_{MSA}^{time}\right)\right)\right) \end{array}\right.$$

(2) $$\left\{\begin{array}{c} X_{MSA}^{dim}=LN_{3}\left(X_{t,:}+Dropout\left(MSA^{dim}\left(X_{t,:},X_{t,:},X_{t,:}\right)\right)\right)\\ X_{out}^{CTCB}=LN_{4}\left(X_{MSA}^{dim}+Dropout\left(MLP^{dim}\left(X_{MSA}^{dim}\right)\right)\right) \end{array}\right.$$

Equation (1) represents the cross-time attention, where MSA is applied to each channel to capture temporal dependencies. Layer Norm (LN1, LN2) is used to normalize features, and Dropout is employed to prevent overfitting. The output of the self-attention module is denoted as $X_{MSA}^{dim}$, which is then followed by an MLP to further extract temporal features. The MLP, equipped with a GELU [37] activation function, serves to capture the nonlinear characteristics of temporal relationships. Throughout the modeling process, skip connections are incorporated to prevent performance degradation. Finally, through Equation (1), the output $X_{out}^{time}$ is obtained, representing the modeled temporal relationships.

After applying Equation (1), we obtain $X_{out}^{time}$, which models the temporal relationships. To further model channel relationships, Equation (2) applies MSA to the multichannel data at each time step. It is important to note that $X_{t,:}$ in Equation (2) is the same as $X_{out}^{time}$. To clarify the modeling objective, we use $X_{t,:}$ to represent the vector of all channels at time step *t*. The modeling process is similar to Equation (1). After applying MSA, an MLP is introduced to capture nonlinear relationships. Similarly, skip connections are incorporated into the design. The final output, $X_{out}^{CTCB}$, is obtained after the two-stage modeling process.

After two steps, the CTCB accomplishes the feature extraction and fusion across time and channels. The CTCB takes inputs from CP as well as the output of the previous CTCB and through the stacking of multiple CTCBs forms the IAB. Within each layer in the IAB (excluding the first one), neighboring patches spanning w units of time are merged, where *w* denotes the size of the merging window. Merging adjacent patches is aimed at obtaining representations at longer time scales. The entire IAB can be modeled as

(3) $$\left\{\begin{array}{cc} l=0 & :X^{IAB,0}=CP\left(X\right)\\ l=1 & :X^{IAB,1}=CTCB\left(X^{IAB,0}\right)\\ l>1 & :X^{IAB,l}=CTCB\left(ML\left(X^{IAB,l-1}\right)\right) \end{array}\right.$$

where *l* signifies the current CTCB layer, $X^{IAB,l}$ denotes the output of the *l*-th layer, ML(·) is the operation of layer merging, and CP(·) is Channel-Patching.

(4) $$\left\{\begin{array}{c} X^{M,l}=X_{t,c}^{M,l},1\leq t\leq \frac{P_{l-1}}{w},1\leq c\leq C\\ X_{t,c}^{M,l}=Linear\left(M\left[X_{wt-w+1,c}^{IAB,l-1},X_{wt,c}^{IAB,l-1}\right]\right) \end{array}\right.$$

For the merge layer, the number of patches in the current layer is initially assessed for divisibility by *w*, with padding applied if necessary. After this, every *w* patches are concatenated, and the resulting concatenation is projected back to the original embedding channel using a linear layer. The merge layer is represented by Equation (4), where $P_{l-1}$ signifies the number of patches in the (l−1)-th layer and $X^{M,l}$ represents the output of the merging operation in the *l*-th layer. $X_{t,c}^{M,l}$ represents the patch in channel *c* at time step *t*. $X_{t,c}^{IAB,l-1}$ represents the patches in channel *c* at time step *t* from the (l−1)-th layer of IAB. M[a,b] denotes the direct concatenation of all patches from *a* to *b*, inclusive of both *a* and *b*. Linear represents the linear layer, which projects the concatenated vector back to the original embedding dimension.

It is worth noting that if we have a total of *H* layers of CTDBs, the actual output of the IAB consists of H+1 layers. This is because in addition to the *H* layers of CTDBs, it also includes $X^{IAB,0}$, which represents the original input passed only through CP.

#### 3.3.2. Channel-Independent Attention Branch

The structure of the CIAB closely resembles that of the IAB, with the primary distinction being that the CIAB maintains channel independence throughout to capture temporal dependency effectively.

**Figure 5:**
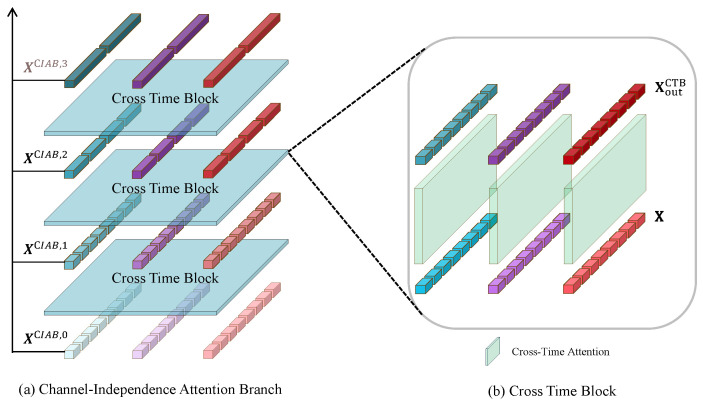
Interlaced Attention Branch.

As illustrated in Figure 5, the CIAB consists of multiple Cross Time Blocks (CTBs) stacked together. Within the CTBs, Multihead Self-Attention (MSA) is solely applied across time. Thus, CTBs can be conceptualized as follows:

(5) $$\left\{\begin{array}{c} X_{MSA}^{time}=LN_{1}\left(X_{:,c}+Dropout\left(MSA^{time}\left(X_{:,c},X_{:,c},X_{:,c}\right)\right)\right)\\ X_{out}^{CTB}=LN_{2}\left(X_{MSA}^{time}+Dropout\left(MLP^{time}\left(X_{MSA}^{time}\right)\right)\right) \end{array}\right.$$

Similar to the IAB, the CIAB also incorporates merging to obtain representations of longer time scales. The CIAB can be modeled as

(6) $$\left\{\begin{array}{cc} l=0 & :X^{CIAB,0}=CP\left(X\right)\\ l=1 & :X^{CIAB,1}=CTB\left(X^{CIAB,0}\right)\\ l>1 & :X^{CIAB,l}=CTB\left(ML\left(X^{IAB,l-1}\right)\right) \end{array}\right.$$

In Equation (6), $X^{CIAB,l}$ denotes the output of the *l*-th layer of CIAB. Similarly, CIAB will output H+1 layers, where $X^{CIAB,0}=CP\left(X\right)$. The IAB and CIAB at the same layer constitute the layers of multiscale dual-branch attention. The modeling of the MDBA is expressed as follows:

(7) $$H^{l}=X^{CIAB,l}⊕X^{IAB,l}$$

$H^{l}$ represents the *l*-th layer of MDBA, and ⊕ indicates that CIAB and IAB act independently, without affecting each other.

### 3.4. Multiscale Classification Head

**Figure 6:**
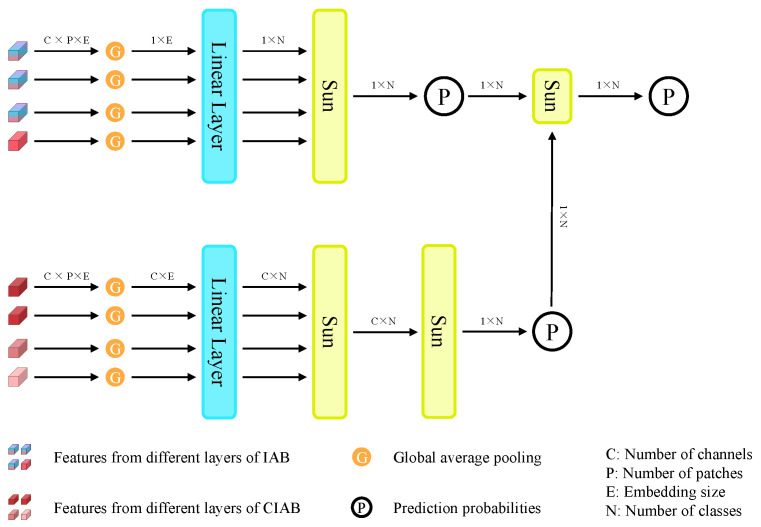
Multiscale Classification Head.

In the Multiscale Classification Head (MCH), the output from the MDBA is received. Different processing methods are applied to the outputs from the IAB and CIAB, as illustrated in Figure 6.

For the IAB, MCH receives the output from H+1 layers and undergoes a linear transformation process where each layer’s feature vectors are projected into *N* dimensions, denoting predicted probability for each layer, where *N* represents the number of categories in the classification task. Then, the predicted probabilities from each layer are summed up to obtain a 1×N vector, representing the predicted probabilities by all layers in the IAB. Note that “Sum” represents addition, but we perform a corresponding averaging operation, i.e., dividing the sum by the number of layers. The same averaging operation is applied to all subsequent “Sum” operations.

For the CIAB, the process is slightly different. After passing through the linear layer, we obtain an (H+1)×C×N vector. Because we maintain channel independence throughout, every channel in each layer of the CIAB produces a prediction probability. For each channel, we sum and average the prediction probabilities across all layers to obtain a C×N vector. Then, we sum and average the probabilities for each channel to obtain a 1×N vector, which represents the prediction probability by the CIAB. Finally, we sum and average the probabilities obtained from both the IAB and CIAB to output the final class probabilities $\hat{y}$.

## 4. Experiment

We evaluated the performance of MD-Former on the UEA [17] and compared it with baseline models. All our experiments were conducted on a computational system using the PyTorch framework. Our computational system is equipped with an NVIDIA GeForce RTX 3090 GPU (24GB).

### 4.1. Experiment Setting

In this section, we elaborate on the specific details of the experiments, including the dataset, parameter settings, and evaluation metrics.

#### 4.1.1. Dataset

The UEA archive comprises 30 real-world multivariate time series datasets, sourced from a wide range of real-life scenarios including human activity recognition, motion classification, and electrocardiogram/electroencephalogram classification. The number of channels varies from 2 to 1345 channels, and the lengths of the time series range from 8 to 17,984. The training sizes of the datasets also vary from 12 to 25,000. Table 1 presents all the information collected by UEA, where “Length” as N/A indicates varying sample lengths. We compared our method with others using the original datasets to evaluate performance fairly. The “Type” field categorizes datasets by their application domains and data modalities:

MOTION: Motion capture data.

ECG: Electrocardiogram recordings.

HAR: Human activity recognition.

AUDIO: Raw audio waveforms or time–frequency representations.

EEG: Electroencephalogram recordings.

OTHER: Specialized sensor data.

SOUND: Acoustic feature sequences.

SPEECH: Speech recognition datasets.

#### 4.1.2. Parameter Setting

We used the Adam optimizer and implemented an early stopping strategy based on the validation set loss, with cross-entropy loss serving as the model’s objective function. To select hyperparameters, we followed the approach outlined in [35]. For each dataset, we randomly split the training set into two parts, 80%–20%, and used the 20% portion as the validation set for hyperparameter tuning. After adjusting the hyperparameters, we trained the model again using the entire training set and evaluated it on the official test set.

#### 4.1.3. Evaluation Metrics

We used accuracy (acc) as the evaluation metric for the classification tasks. Considering the large number of datasets and baseline models, it is not feasible for a single model to achieve the best performance on all tasks. Therefore, we also provided aggregated statistics, including average accuracy (AVG acc), average rank (AVG rank), number of wins (win), and Mean Per-Class Error (MPCE).

AVG acc/AVG rank: On the 30 datasets from the UEA archive, we considered the average accuracy and average rank across all datasets to reflect the method’s generalization performance.

Win: It represents the number of times the method achieved wins across the 30 datasets, reflecting the comprehensive performance of the method. However, it may not provide absolute reliability in assessing comprehensive performance.

MPCE: The MPCE index is an effective method for calculating the average error rate considering the number of categories [38]. The formula is as follows:

(8) $$MPCE=\frac{1}{D}\sum_{d=1}^{D}\frac{e_{k}}{D_{k}}$$

where *D* is the number of datasets, $D_{k}$ represents the number of categories in the *k*-th dataset, and $e_{k}$ denotes the error rate of the current method on the *k*-th dataset. A smaller MPCE value indicates better performance of the method.

The MPCE index is an effective method for calculating the average error rate while considering the number of categories in each dataset. This ensures that datasets with a larger number of categories do not disproportionately influence the final performance evaluation. By normalizing the error rate $e_{k}$ of each dataset *k* based on its number of categories $D_{k}$, MPCE balances the impact of each category. A smaller MPCE value indicates better performance, as it reflects the model’s ability to maintain a low error rate across various datasets. MPCE is particularly suitable for multiclass classification problems, providing a unified evaluation standard for datasets with varying numbers of categories and mitigating the issue of error rate distortion caused by class imbalance.

### 4.2. Comparison Method

We compared MD-Former with the following methods:

1. Distance-based method: Euclidean Distance (ED), dimension-dependent dynamic time warping (DTWI), and dimension-dependent dynamic time warping (DTWD) [39]. These methods rely on manually designed similarity metrics, which may not adapt well to the complexity of different datasets. Furthermore, as the dimensionality of the data increases, the computational complexity and storage requirements grow sharply, limiting their application on high-dimensional data.
2. WEASEL-MUSE [40] is a bag-of-pattern-based sliding-window approach with statistical feature extraction and filtration. Although statistical feature extraction helps to reduce the dimensionality, this method may overlook the details of temporal information, leading to information loss, especially in high-dimensional data. Moreover, performing symbolization independently for each variable results in the feature dimensionality increasing linearly with the number of variables.
3. MLSTM-FCNs [11] extend LSTM-FCN and Attention LSTM-FCN by adding squeeze and excitation blocks. Despite the addition of compression and activation modules, LSTM networks still face the issues of vanishing and exploding gradients, especially when handling long sequences, which may prevent them from effectively capturing long-term dependencies.
4. Rocket [41] convolves time series with random convolutional kernels and applies global max pooling to extract features. Although the use of random convolutional kernels can effectively extract features, this method lacks contextual understanding of time series data, which may lead to instability in feature extraction. Additionally, it relies on global max pooling, which may result in the loss of important local information.
5. TapNet [33] introduces attention prototype networks into MTSC and extends them to semi-supervised learning by leveraging unlabeled data. The integration of multiple deep learning methods results in a higher model complexity, which may lead to longer training times. Additionally, it lacks sufficient cross-channel and multiscale information.
6. ShapeNet [10] extends a shapelet to multivariate time series. It learns shared embedding space across different shapelet candidates and trains a dilated causal CNN followed by an SVM. Assuming that the features of time series data can be unified through a shared embedding space may lead to the model’s insufficient ability to represent certain complex patterns. Additionally, selecting partial shapelets to represent the original data may result in information loss.
7. ConvTran [34] explores the role of positional encoding in a Transformer on MTS, improving the positional encoding and data embedding of time series data. However, it lacks multiscale perception of time series data and does not have cross-channel information interaction.
8. Time Series Transformer (TST) [35] designs a pretraining task of randomly masking time segments and reconstructing them to enhance the model’s representation ability of the data. Directly introducing a Transformer into the time series domain lacks unique designs tailored for time series data, such as multiscale modeling.
9. TARNet [36] uses a Transformer to learn task-aware data reconstruction, aiming to enhance the model’s representation ability for downstream tasks. Further improvements to TST enhance sensitivity to downstream tasks but at the cost of some generalization performance. Additionally, like TST, it lacks a dedicated model design for time series.

### 4.3. Performance Evaluation

To evaluate the specific performance of MD-Former, we compare it with the methods mentioned in Section 4.2. Some methods only report accuracy on a subset of the UEA archive, so “N/A” in the table results indicates that the method did not report accuracy for the corresponding dataset. The bold numbers represent the best results among all models.

As shown in Table 2, our method achieved nine wins on 30 datasets, comparable to the previous best-performing methods. Moreover, MD-Former exhibited superior performance in terms of AVG rank, AVG acc, and MPCE metrics. Notably, it is imperative to recognize that our proposed methodology does not manifest statistically significant superiority in terms of the win metric performance at elevated accuracy thresholds. Specifically, when analyzing instances where classification accuracy surpasses 0.7, MD-Former achieves a win count (acc > 0.7) of 6, which fails to demonstrate substantial dominance over comparative approaches. However, it must be emphasized that this singular metric inadequately encapsulates the model’s comprehensive capabilities, as the win metric inherently provides a constrained evaluation framework. In contradistinction, the MPCE metric facilitates a more rigorous and multidimensional performance assessment through its sophisticated quantification paradigm. This analytical distinction substantiates the enduring performance superiority of MD-Former when evaluated through holistic evaluation criteria.

Specifically, our method surpassed the leading approach by more than 13 percentage points in accuracy on the StandWalkJump dataset. Noteworthy is the observation that for datasets with lengths surpassing 1500, such as EigenWorms, EthanolConcentration, MotorImagery, and StandWalkJump, we achieved the best performance, indicating the significant effectiveness of multiscale attention for longer datasets. The experimental results demonstrate the outstanding performance of MD-Former, particularly in handling real-world, complex, and longer datasets. MD-Former presents a novel approach for applying the Transformer in MTSC.

**Figure 7:**
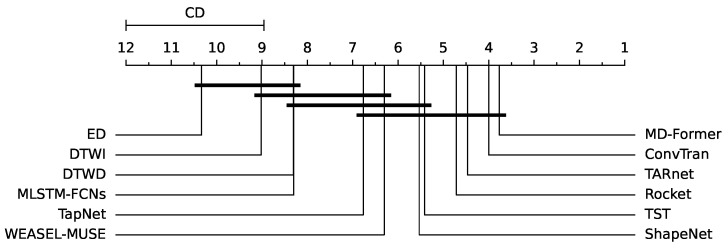
The CD (Critical Difference) plot for 12 multivariate time series classification methods across 30 datasets, with a confidence level of 0.95. Thick horizontal lines indicate groups of methods that do not have a significant difference.

To assess the statistical variances between MD-Former and alternative methodologies across various datasets, we conducted the Nemenyi test based on the “AVG rank” across different datasets. Consistent with prior research [12], a Critical Difference (CD) plot was generated to visually represent the distinctions. The CD plot arranges the 12 multivariate time series classifiers along a horizontal line based on their “AVG rank”. As shown in Figure 7, MD-Former demonstrates the best overall performance across different datasets, highlighting its strong robustness across various scenarios.

### 4.4. Ablation Study

In Section 4.3, we demonstrated the feasibility of MD-Former for MTSC. In this section, we conducted ablation experiments to evaluate the effectiveness of the proposed modules by removing the main design modules of MD-Former. The ablation experiments were conducted on 30 datasets, and the experimental settings followed those outlined in Section 4.1. Table 3 presents the comparisons from the ablation experiments.

We designed ablations of MD-Former, “MD_wo_IAB”, “MD_wo_CIAB”, and “MD_wo_MS”. The ablations “MD_wo_IAB” and “MD_wo_CIAB” remove the IAB and CIAB from MD-Former, respectively. The “MD_wo_MS” ablation removes the multiscale design, replacing it with a single-layer attention network after Channel-Patching, followed by the classification head for result generation. It is noteworthy that MD-Former has H+1 layers of multiscale attention. To maintain “MD_wo_MS” as a single-layer structure, we removed the $H^{0}$ and only used $H^{1}$ for classification because $H^{0}$ does not undergo an attention mechanism.

#### 4.4.1. IAB and CIAB

We investigated the impact of different branches on the model, as illustrated in Table 3. In comparison to “MD_wo_IAB” and “MD_wo_CIAB”, “MD_wo_CIAB” demonstrates superior classification performance, particularly evident in Human Activity Recognition (HAR) datasets. HAR datasets typically use sensors to record human activities, such as different values in the X, Y, and Z directions recorded by accelerometers and gyroscopes. It is evident that real-world human activities involve synchronous movement in the X, Y, and Z directions. Therefore, on HAR datasets, the correlation between different channels is significant, and cross-channel information fusion is crucial for the model to exploit channel dependencies. “MD_wo_CIAB” effectively fuses cross-channel features, leading to a significant improvement in classification performance.

Furthermore, we compared “MD_wo_CIAB” and MD-Former to confirm the significance of channel independence. Although channel independence did not result in a significant performance boost, the overall performance of MD-Former improved with the introduction of the channel-independent branch. Specifically, on the EigenWorms dataset, classification accuracy increased by approximately 7%. EigenWorms is a worm motion dataset where six channels represent the amplitude along six directions. A design that only performs channel fusion might confuse the amplitude information along each direction, which is crucial for classification. Introducing the channel-independent branch helps independently extract information from each direction. Furthermore, the EigenWorms dataset has a data length of 17,984, and we set large-scale patching and deeper network layers in the model. In the IAB, as the number of layers increases, the frequent fusion of channel and time information may lead to confusion and loss of information, whereas the CIAB avoids excessive fusion of channel and time information.

By comparing the IAB and CIAB branches, we explore the theoretical impact of the dual-branch architecture on classification tasks. The IAB alone appears to be quite effective, as it already performs fusion and extraction of both time and channel information. However, introducing the CIAB not only improves overall performance but also leads to significant gains on specific datasets, as demonstrated in Table 3. The key advantage of the CIAB is that it preserves the integrity of the time relationship by avoiding excessive interference from channel information. In time series data, time information is typically the most critical, and introducing channel information can sometimes disrupt this relationship. The purpose of the CIAB is to maintain the purity of the time-related features within the multilayer architecture. In summary, while the IAB serves as the core of the network, the CIAB enriches the network by providing additional details on time-related information, ensuring that the time relationship remains the dominant feature.

Finally, we investigate the potential negative impacts of introducing the IAB and CIAB on certain datasets. For instance, on the PhonemeSpectra dataset, “MD_wo_IAB” achieves the best performance, while the inclusion of IAB leads to a decrease in accuracy. However, it is worth noting that the overall accuracy remains relatively low, with the best-performing model, ConvTran, achieving only 0.306 accuracy. This suggests that generic time series models may inherently struggle with the PhonemeSpectra dataset, and the observed differences do not indicate significant methodological issues.

Furthermore, regarding the CIAB, we observe that on datasets such as NATOPS, PEMS-SF, and PenDigits, the standalone IAB performs well, while the introduction of the CIAB results in degraded performance, particularly on the NATOPS dataset. We analyze this phenomenon and identify that NATOPS and PEMS-SF share two distinct characteristics: shorter sequence lengths and higher channel dimensions. This implies that interchannel relationships may play a more critical role than temporal dependencies in these datasets. When the CIAB is introduced, it operates exclusively on the temporal dimension, thereby increasing the model’s focus on temporal patterns while potentially reducing its attention to channel-wise interactions. This shift in focus may negatively impact the modeling of channel-dominant data.

Nevertheless, as previously discussed, the CIAB demonstrates clear advantages and contributes to overall performance improvements. Given that MD-Former is designed as a general-purpose framework, we believe that the inclusion of the CIAB is justified, as it enhances the model’s adaptability and effectiveness across diverse datasets.

#### 4.4.2. Multiscale Architecture

In our Multiscale Dual-Branch Attention, we employed a multiscale architecture, where both the IAB and CIAB have multiple layers, to capture relationships at different scales. An ablation experiment, labeled as “MD_wo_MS”, was conducted to assess the efficacy of this multiscale architecture.

Specifically, MD-Former outperformed “MD_wo_MS” on datasets with data lengths exceeding 150. The increase in data length indicates a rise in the complexity of temporal relationships, where different time scales are beneficial for uncovering time relationships across various patterns and periodic behaviors. Thanks to the multiscale architecture, MD-Former can extract cross-channel and cross-temporal relationships at different scales, facilitating the discovery of periodic patterns across scales. Of course, the multiscale design introduces more parameters, but considering the performance gains, we believe this efficiency trade-off is worthwhile. Additionally, we designed CP and merge layers to adjust the scales and reduce the parameter count.

### 4.5. Visual Analysis on the Attention Module

To demonstrate the efficacy of MD-Former in extracting multiscale, cross-channel, and cross-temporal information, we provide real-world examples. Based on the Multiscale Dual-Branch Attention described in Section 3.3, we compute and visualize the normalized attention scores.

#### 4.5.1. Case 1: Time and Channel Dependencies

**Figure 8:**
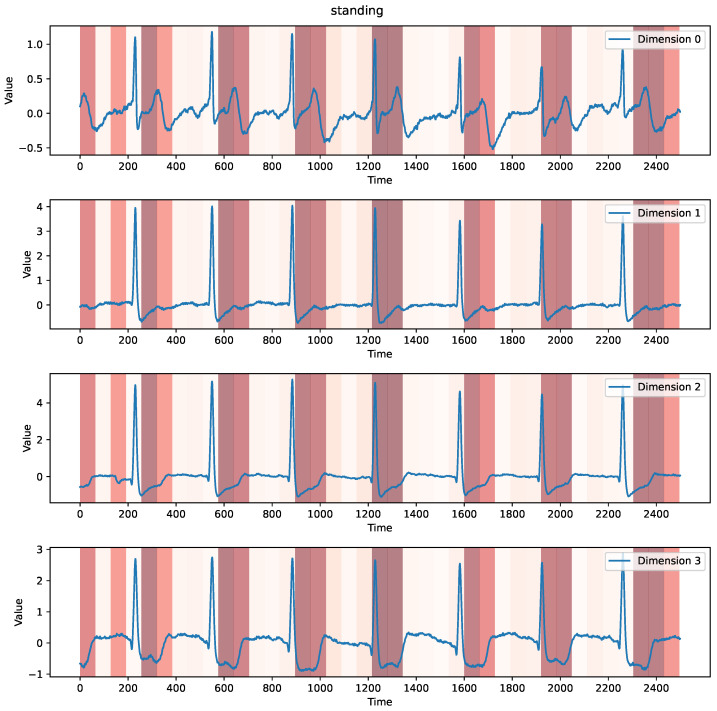
A “standing” instance from the StandWalkJump dataset. The blue lines represent the actual data, with each line corresponding to a different channel (4 channels in total). The background color represents the attention scores of the last layer in the IAB, with darker shades indicating higher scores.

Figure 8 illustrates an example from the StandWalkJump dataset, which comprises electrocardiogram (ECG) signals recorded from a 25-year-old healthy male performing different physical activities (standing, walking, jumping). The data length is 2500. As is evident in Figure 8, the instance exhibits periodicity in all channels. Notably, the attention scores also exhibit periodicity, demonstrating that MD-Former effectively captures the inherent periodicity within the data.

Furthermore, it is interesting to note that the peaks of attention scores seem to lag slightly behind the peaks of the original data. However, upon closer observation of Figure 8, it can be noticed that there are significant differences in the data across the four channels at the peaks of attention scores. We attribute the delay in the peaks of attention scores to MD-Former’s ability to capture cross-channel information. For instance, from a global perspective, there are no regular changes at the start and end of the data, yet the attention scores remain relatively high at these positions. This is because, at the beginning and end, the original data exhibit significant differences across channels. This example demonstrates that modeling the channel relationships directly impacts the model’s feature extraction. By applying the attention mechanism across channels, the model can identify variation patterns across different channels, enhancing its understanding and exploration of the relationships between channels.

#### 4.5.2. Case 2: Multiscale Relationships

**Figure 9:**
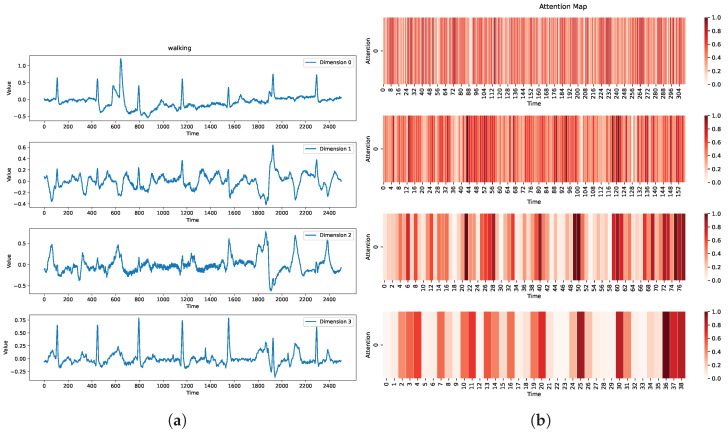
A “walking” instance from the StandWalkJump dataset. (**a**) Original data, which consist of four channels. (**b**) Attention scores from different layers of IAB. Time scale increases from top to bottom.

Figure 9 also presents an instance from StandWalkJump. By analyzing the attention score heatmap, we observe that at smaller scales, attention scores are generally higher. This is because with longer sequences, attention struggles to model global information, and even small fluctuations in local regions can significantly impact attention scores. However, at larger scales, especially in the last layer of the IAB, attention is primarily focused on a few key areas, while the attention scores in most regions are lower. Although we cannot fully analyze the specific areas of focus at larger scales, it is clear that the attention pattern shifts from being dispersed across local regions to concentrating on a few key areas. We hypothesize that these concentrated areas at larger scales may contain latent information, such as varying trends across different channels. This may be because, at larger scales, the model can observe more information, thereby revealing global dependencies within the data. We interpret this change in attention scores as an indication that as the scale increases, the model becomes more effective at identifying globally important data points. This example demonstrates the impact of the multiscale architecture on the model. Larger scales provide a broader field of view, allowing the model to perceive important data points in the global context. Meanwhile, smaller scales help the model capture local trends and understand local information.

### 4.6. Empirical Evaluation of Efficiency and Effectiveness

To evaluate the model’s efficiency and effectiveness, we measured its training time. ConvTran was chosen as a baseline for efficiency comparison due to its remarkable performance, achieved by avoiding multiscale design and utilizing convolution as a preprocessing network to reduce complexity. We selected several representative datasets for evaluation: EigenWorms (the dataset with the longest sequence length), EthanolConcentration (a dataset with relatively long sequences), PEMS-SF (a dataset with a large number of channels), and PenDigits (a dataset with a large training set). To ensure a fair comparison, all models were trained for 50 epochs, and both training time and accuracy were recorded. For this evaluation, our computational platform was equipped with an RTX 4090 (24 GB).

As shown in Table 4, on the EigenWorms dataset, even when we attempted to train the ConvTran model with the smallest batch size, it still exceeded the GPU memory limit, so no results are reported for this dataset. From the table, it is evident that MD-Former excels on datasets with longer sequences, achieving higher classification accuracy than ConvTran while maintaining competitive efficiency. This advantage is primarily attributed to the Channel-Patching, which alleviates the exponential growth in parameters caused by long sequences.

For datasets with a large number of channels, MD-Former does not exhibit an advantage in training time. For instance, on the PEMS-SF dataset, the training time is considerably longer. This is because MD-Former incorporates channel relationship modeling and features a channel-independent branch, both of which increase the model’s complexity, resulting in slower training. Nevertheless, the accuracy improvement is remarkable. On the PEMS-SF dataset, MD-Former outperforms ConvTran by nearly 20 percentage points. We believe this trade-off in efficiency is justified. It is also worth noting that across the UEA dataset, only a few datasets have more than 10 channels, indicating that an excessively high number of channels is rare in real-world scenarios.

Moreover, on datasets with larger training scales, ConvTran demonstrates certain advantages in training time. While MD-Former achieves comparable final accuracy to ConvTran (as shown in Table 2) and generally trains faster, ConvTran converges more quickly, reaching its optimal accuracy within 50 epochs, whereas MD-Former has yet to achieve its best performance at that point. Balancing training time and accuracy, ConvTran’s training time for 50 epochs on the PenDigits dataset is nearly five times longer than that of MD-Former, with only a minor accuracy difference. This underscores MD-Former’s excellent overall performance in terms of both efficiency and accuracy.

### 4.7. Discussion

MD-Former, through its unique embedding design, adapts to time series data, effectively preserving the original information while significantly reducing the computational complexity of the subsequent Transformer. Additionally, we believe that the patching method actually provides local perception of the data. Modeling local information into a patch is essentially a process of small receptive fields. Furthermore, we strengthened the interaction across time and channels, which are the main features of multivariate time series—time relationships and channel interactions. Time relationships are inherent properties of time series data, and this is a crucial aspect that the model must focus on. Channel relationships, on the other hand, have shown significant importance in recent literature, especially in datasets where channel relationships are prominent. Finally, in order to capture periodic patterns over time, we designed a multiscale architecture, as periodic relationships are apparent in the real world and reveal the cyclical laws of the physical world. Through the construction of MD-Former, we believe that the main idea for designing a multivariate time series model is to align with the characteristics of time series data, adopting a data-driven approach. Our focus is primarily on data embedding, time and cross-channel modeling, and multiscale design. This provides a fundamental approach for future multivariate time series models.

However, in practice, the Channel-Patching design may face an issue: it does not fully adapt to variable-length data. Truncation or padding may lead to information loss, which is one of the key directions for our future work. Additionally, in the feature extraction module, balancing time relationships and channel interactions will be another focus for the future. An imbalance in modeling one relationship can inevitably lead to the insufficient modeling of the other. Similarly, there is also an imbalance in the parameter volume between feature extraction and feature fusion. We have also pointed out the limitations of MD-Former in certain scenarios, such as imperfect performance when the number of channels is too large. We are considering introducing an adaptive parameter balancing mechanism in future work to enhance sensitivity to downstream tasks. We hope that MD-Former will introduce new ideas and benchmarks for the MTSC problem.

## 5. Conclusions

In conclusion, this paper introduces the Multiscale Dual-Branch Attention mechanism, comprising the IAB and CIAB, which constructs a multiscale feature extraction architecture. By fully leveraging dependencies across multiple scales, channels, and time steps, this mechanism significantly enhances feature extraction capabilities. Additionally, the introduction of Channel-Patching not only reduces complexity but also facilitates the capture of longer-scale dependencies.

Experimental results demonstrate the efficacy of MD-Former, showcasing competitive performance with state-of-the-art models on the UEA archive and surpassing them on long-sequence datasets. However, we also acknowledge some limitations, particularly the imbalance in the parameter volume between feature extraction and feature fusion, the simplicity of the classification head structure that does not fully utilize the features, and the suboptimal efficiency performance on datasets with a large number of channels.

Moving forward, future research will focus on optimizing the parameter count of the backbone network, introducing new feature fusion modules, and designing novel classification heads to achieve more comprehensive information fusion and more efficient feature modeling. These efforts aim to further enhance performance. 

## Figures and Tables

**Table 1:** Overview of the 30 datasets in the UEA archive (Abbrev. = Abbreviation; Dim. = Dimensions; Train/Test = Number of samples).

Dataset	Character						
	Abbrev.	Train	Test	Dim.	Length	Classes	Type
ArticularyWordRecognition	AWR	275	300	9	144	25	MOTION
AtrialFibrillation	AF	15	15	2	640	3	ECG
BasicMotions	BM	40	40	6	100	4	HAR
CharacterTrajectories	CT	1422	1436	3	N/A	20	MOTION
Cricket	CR	108	72	6	1197	12	HAR
DuckDuckGeese	DDG	50	50	1345	270	5	AUDIO
EigenWorms	EW	128	131	6	17,984	5	MOTION
Epilepsy	EP	137	138	3	206	4	HAR
ERing	ER	30	270	4	65	6	HAR
EthanolConcentration	EC	261	263	3	1751	4	OTHER
FaceDetection	FD	5890	3524	144	62	2	EEG
FingerMovements	FM	316	100	28	50	2	EEG
HandMovementDirection	HMD	160	74	10	400	4	EEG
Handwriting	HW	150	850	3	152	26	HAR
Heartbeat	HB	204	205	61	405	2	AUDIO
InsectWingbeat	IW	25,000	25,000	200	N/A	10	AUDIO
JapaneseVowels	JV	270	370	12	29	9	AUDIO
Libras	LIB	180	180	2	45	15	HAR
LSST	LSST	2459	2466	6	36	14	OTHER
MotorImagery	MI	278	100	64	3000	2	EEG
NATOPS	NA	180	180	24	51	6	HAR
PEMS-SF	PEMS	267	173	963	144	7	OTHER
PenDigits	PD	7494	3498	2	8	10	MOTION
PhonemeSpectra	PS	3315	3353	11	217	39	SOUND
RacketSports	RS	151	152	6	30	4	HAR
SelfRegulationSCP1	SRS1	268	293	6	896	2	EEG
SelfRegulationSCP2	SRS2	200	180	7	1152	2	EEG
SpokenArabicDigits	SAD	6599	2199	13	93	10	SPEECH
StandWalkJump	SWJ	12	15	4	2500	3	ECG
UWaveGestureLibrary	UW	120	320	3	315	8	HAR

**Table 2:** Performance comparison of MD-Former and baselines on UEA archive.

**Dataset**	**Methodology**					
	**ED**	**MLSTM-FCNs**	**WEASEL-MUSE**	**DTWI**	**Rocket**	**ConvTran**
**AWR**	0.970	0.973	0.990	0.980	**0.993**	0.983
**AF**	0.267	0.267	0.333	0.267	0.067	0.400
**BM**	0.676	0.950	**1.000**	**1.000**	**1.000**	**1.000**
**CT**	0.964	0.985	0.990	0.969	0.991	0.992
**CR**	0.944	0.917	**1.000**	0.986	**1.000**	**1.000**
**DDG**	0.275	0.675	0.575	0.550	0.500	0.620
**EW**	0.549	0.504	0.890	N/A	0.650	0.593
**EP**	0.666	0.761	**1.000**	0.978	0.986	0.986
**ER**	0.133	0.133	0.133	0.133	**0.989**	0.963
**EC**	0.293	0.373	0.430	0.304	0.450	0.361
**FD**	0.519	0.545	0.545	N/A	0.638	**0.672**
**FM**	0.550	0.580	0.490	0.520	0.520	0.560
**HMD**	0.278	0.365	0.365	0.306	0.486	0.405
**HW**	0.200	0.286	**0.605**	0.316	0.596	0.375
**HB**	0.619	0.663	0.727	0.658	0.741	0.785
**IW**	0.128	0.167	N/A	N/A	0.179	**0.713**
**JV**	0.924	0.976	0.973	0.959	0.978	0.989
**LIB**	0.833	0.856	0.878	0.894	0.906	0.928
**LSST**	0.456	0.373	0.590	0.575	0.635	0.615
**MI**	0.510	0.510	0.500	N/A	0.460	0.560
**NA**	0.850	0.889	0.870	0.850	0.872	**0.944**
**PEMS**	0.705	0.699	N/A	0.734	0.832	0.828
**PD**	0.973	0.978	0.948	0.939	0.981	0.987
**PS**	0.104	0.110	0.190	0.151	0.273	**0.306**
**RS**	0.868	0.803	0.934	0.842	0.901	0.862
**SRS1**	0.771	0.874	0.710	0.765	0.867	0.920
**SRS2**	0.483	0.472	0.460	0.533	0.555	0.583
**SAD**	0.967	0.990	0.982	0.959	0.997	N/A
**SWJ**	0.200	0.067	0.333	0.333	0.467	0.333
**UW**	0.881	0.891	0.916	0.868	**0.931**	0.891
**Win**	0	0	4	1	5	6
**AVG rank**	10.33	8.30	6.30	9.02	4.72	4.00
**AVG acc**	0.5852	0.6211	0.6913	0.6680	0.7147	0.7294
**MPCE**	0.1005	0.0919	0.0862	0.0784	0.0757	0.0706
**Dataset**	**Methodology**					
	**DTWD**	**TapNet**	**ShapeNet**	**TST**	**TARnet**	**MD-Former**
**AWR**	0.987	0.987	0.987	0.947	0.977	0.983
**AF**	0.220	0.333	0.400	0.533	**1.000**	0.600
**BM**	0.975	**1.000**	**1.000**	0.925	**1.000**	**1.000**
**CT**	0.989	**0.997**	0.980	0.971	0.994	0.990
**CR**	**1.000**	0.958	0.986	0.847	**1.000**	0.986
**DDG**	0.600	0.575	0.725	0.300	**0.750**	0.500
**EW**	0.618	0.489	0.878	0.720	0.420	**0.901**
**EP**	0.964	0.971	0.987	0.775	**1.000**	0.986
**ER**	0.133	0.133	0.133	0.930	0.919	0.952
**EC**	0.323	0.323	0.312	0.337	0.323	**0.460**
**FD**	0.529	0.556	0.602	0.625	0.641	0.653
**FM**	0.530	0.530	0.580	0.590	0.620	**0.650**
**HMD**	0.231	0.378	0.338	**0.675**	0.392	0.446
**HW**	0.286	0.357	0.451	0.359	0.281	0.261
**HB**	0.717	0.751	0.756	0.782	0.780	**0.790**
**IW**	N/A	0.208	0.250	0.687	0.137	0.710
**JV**	0.949	0.965	0.984	**0.995**	0.992	0.978
**LIB**	0.870	0.850	0.856	0.861	**1.000**	0.894
**LSST**	0.551	0.568	0.590	0.576	**0.976**	0.594
**MI**	0.500	0.590	0.610	0.610	0.630	**0.660**
**NA**	0.883	0.939	0.883	0.939	0.911	0.911
**PEMS**	0.711	0.751	0.751	0.930	0.936	**0.960**
**PD**	0.977	0.980	0.977	0.981	0.976	**0.988**
**PS**	0.151	0.175	0.298	0.111	0.165	0.175
**RS**	0.803	0.868	0.882	0.796	**0.987**	0.895
**SRS1**	0.775	0.652	0.782	**0.961**	0.816	0.915
**SRS2**	0.539	0.550	0.578	0.604	**0.622**	0.600
**SAD**	0.963	0.983	0.975	**0.998**	0.985	0.989
**SWJ**	0.200	0.400	0.533	0.600	0.533	**0.733**
**UW**	0.903	0.894	0.906	0.913	0.878	0.897
**Win**	1	2	1	4	**9**	**9**
**AVG rank**	8.31	6.77	5.53	5.42	4.47	**3.77**
**AVG acc**	0.6509	0.6570	0.6990	0.7293	0.7547	**0.7686**
**MPCE**	0.0908	0.0847	0.0742	0.0651	0.0596	**0.0558**

**Table 3:** Performance comparison of MD-Former and baselines on UEA archive.

Dataset	MD-Former	MD_wo_IAB	MD_wo_CIAB	MD_wo_MS
**AWR**	**0.983**	0.957	0.973	0.923
**AF**	**0.600**	0.533	0.467	0.467
**BM**	**1.000**	**1.000**	**1.000**	**1.000**
**CT**	0.990	0.985	**0.991**	0.978
**CR**	**0.986**	0.917	0.944	0.917
**DDG**	**0.500**	0.440	**0.500**	0.480
**EW**	**0.901**	0.771	0.832	0.893
**EP**	**0.986**	0.964	0.964	0.985
**ER**	**0.952**	0.926	**0.952**	0.948
**EC**	**0.460**	0.368	0.335	0.350
**FD**	0.653	0.634	**0.663**	0.650
**FM**	**0.650**	0.610	0.610	0.600
**HMD**	**0.446**	**0.446**	0.432	0.405
**HW**	**0.261**	0.168	0.248	0.171
**HB**	**0.790**	0.785	0.771	0.732
**IW**	0.710	0.680	**0.711**	0.670
**JV**	**0.978**	0.932	0.976	0.965
**LIB**	**0.894**	0.856	**0.894**	0.861
**LSST**	0.594	0.403	**0.622**	0.504
**MI**	**0.660**	0.650	0.630	0.650
**NA**	0.911	0.856	**0.950**	0.861
**PEMS**	0.960	0.936	**0.979**	0.977
**PD**	**0.988**	0.981	0.982	0.984
**PS**	0.175	**0.191**	0.173	0.165
**RS**	**0.895**	0.849	0.868	0.822
**SRS1**	**0.915**	0.881	0.891	0.867
**SRS2**	**0.600**	0.594	0.594	0.578
**SAD**	**0.989**	0.976	0.983	0.959
**SWJ**	**0.733**	0.533	**0.733**	0.467
**UW**	**0.897**	0.859	0.878	0.863
**Win**	**23**	3	11	1
**AVG acc**	**0.769**	0.723	0.752	0.723
**MPCE**	**0.0558**	0.0652	0.0610	0.0669

**Table 4:** Comparison of runtime and accuracy between ConvTran and MD-Former.

Dataset	TranSize	Dimensions	Length	ConvTran		MD-Former	
				Accuracy	Train Time	Accuracy	Train Time
EW	128	6	17984	N/A	N/A	0.901	60.462
EC	261	3	1751	0.293	47.121	0.433	38.828
PEMS	267	963	144	0.723	189.988	0.933	561.664
PD	7494	2	8	0.987	367.828	0.968	80.910

## Data Availability

The data used in this study are publicly available at https://www.timeseriesclassification.com/dataset.php (accessed on 15 January 2024).
